# Analysis of clinical outcomes of intra-aortic balloon pump use during coronary artery bypass surgery

**DOI:** 10.5830/CVJA-2015-010

**Published:** 2015

**Authors:** Gunduz Yumun, Ufuk Aydin, Yusuf Ata, Faruk Toktaş, Arda Aybars Pala, Ahmet Fatih Ozyazicioglu, Tamer Turk, Senol Yavuz

**Affiliations:** Department of Cardiovascular Surgery, Namik Kemal University, Tekirdag, Turkey; Department of Cardiovascular Surgery, Bursa Yuksek Ihtisas Education and Research Hospital, Bursa, Turkey; Department of Cardiovascular Surgery, Bursa Yuksek Ihtisas Education and Research Hospital, Bursa, Turkey; Department of Cardiovascular Surgery, Bursa Yuksek Ihtisas Education and Research Hospital, Bursa, Turkey; Department of Cardiovascular Surgery, Bursa Yuksek Ihtisas Education and Research Hospital, Bursa, Turkey; Department of Cardiovascular Surgery, Bursa Yuksek Ihtisas Education and Research Hospital, Bursa, Turkey; Department of Cardiovascular Surgery, Bursa Yuksek Ihtisas Education and Research Hospital, Bursa, Turkey; Department of Cardiovascular Surgery, Bursa Yuksek Ihtisas Education and Research Hospital, Bursa, Turkey

**Keywords:** intra-aortic balloon pump, coronary artery bypass, mortality

## Abstract

**Aim:**

The mortality rate of coronary artery bypass surgery increases with advanced patient age. This intra-aortic balloon pump (IABP) study was conducted to compare older patients (above 65 years of age) with younger patients (below 65 years of age) who had undergone coronary artery bypass surgery and had had an IABP inserted, with regard to hospital stay, clinical features, intensive care unit stay, postoperative complications, and mortality and morbidity rates.

**Methods:**

One hundred and ninety patients who had undergone coronary artery bypass surgery and had required IABP support were enrolled in this study. Patients younger than 65 years of age were considered younger, and the others were considered older. Ninety-two patients were in younger group and 98 patients were older group. The mortality rates, pre-operative clinical characteristics, postoperative complications, and duration of intensive care unit and hospital stay of the groups were compared. The risk factors for mortality and complications were analysed.

**Results:**

One hundred and thirty-eight of the patients were male, and the mean age was 62.7 ± 9.9 years. The mortality rate was higher in the older patient group than the younger group [34 (37.7%) and 23 (23.4 %), respectively (*p* = 0.043)]. The crossclamp time, mean ejection fraction, cardiopulmonary bypass time, and length of stay in the intensive care unit were similar between the two groups (*p* > 0.05). Cardiopulmonary bypass time was the unique independent risk factor for mortality in both groups.

**Conclusion:**

In this study, high mortality rates in the postoperative period were similar to those in prior studies regarding IABP support. The complication rates were higher in the older patient group. Prolonged cardiopulmonary bypass time and advanced age were determined to be significant risk factors for mortality.

## Aim

An intra-aortic balloon pump (IABP) increases coronary blood flow and reduces left ventricular afterload.^1^-^3^ It helps to increase the required amount of time for the heart to recover in low-cardiac output syndrome following a cardiopulmonary bypass (CPB) or ischaemic events. In earlier reports, researchers had suggested that postoperative heart failure was the single indication for IABP support.^1^,^2^ However, these indications have widened, and the use of IABP support has recently become more common.

Frequently reported complications of IABP include bleeding, aorto-iliac injury and thrombocytopaenia.^4^,^5^ In-hospital mortality and the early mortality of patients requiring IABP support is high, ranging from 26 to 50%, due to the cardiac problems that initially led to the need for this support.^6^,^7^

The size of elderly population has been continuously increasing across the globe. Parallel with this increase, the number of older patients being referred for coronary artery bypass grafting (CABG) has also increased.^8^ Although several studies have shown a significant increase in surgical mortality rates of elderly patients,^9^ there have been no studies regarding clinical outcomes of IABP use in elderly patients.

In this study, we aimed to compare older patients with younger patients regarding clinical features, postoperative complications, intensive care unit and hospital stays, and morbidity and mortality rates in patients who had undergone coronary artery bypass surgery and required IABP support.

## Methods

Patients who had undergone CABG in our clinic between 2008 and 2013 were retrospectively evaluated. Patients who had undergone combined CABG and heart valve surgery were excluded. This study was granted the full approval of the institutional Review Board.

Three hundred and eighty-eight (7.4%) of 4 940 consecutive patients had required IABP support following CABG. Among these patients, IABP was used intra-operatively for 190 patients. One hundred and thirty-eight of the patients were male, and the mean age was 62.7 ± 9.9 years. The demographic characteristics of the patients are summarised in Table 1.

**Table 1:** Demographic characteristics of the patients

	*Younger group (n = 98)*	*Older group (n = 92)*	*Total*	*p-value*
Male/female	74/24	64/28	138/52	0.358
Mean age	54.7 ± 6.1	71.4 ± 4.5	62.7 ± 9.9	< 0.001
Mean EF	37.1 ± 8.3	39.2 ± 9.5	38.1 ± 8.9	0.121
MI, *n* (%)	31 (31.9)	24 (26)	55 (27.7)	0.400
COPD, *n* (%)	5 (5.1)	13 (14.1)	18 (9)	0.034
CRF, *n* (%)	3 (3)	5 (5.4)	8 (4.2)	0.487
Redo, *n* (%)	3 (3)	0	3 (1.5)	0.297
HT, *n* (%)	47 (48)	56 (60)	103 (54)	0.074
DM, *n* (%)	48 (49)	23 (25)	71 (37.3)	0.001
CVA, *n* (%)	4 (4.1)	5 (5.4)	9 (4.7)	0.745
Recent MI, *n* (%)	18 (18.3)	16 (17.4)	34 (17.9)	0.861
EuroSCORE	4 (0–10)	5 (2–10)	4 (0–10)	< 0.001
BMI	27.2 ± 4	26.7 ± 4.4	27.2 ± 4.1	0.112
LMCA	8 (8.1)	5 (5.4)	13 (6.8)	0.457
Prophylactic levosimendan	18 (18.3)	12 (13)	30 (15.8)	0.315
Emergency	18 (18.3)	16 (17.4)	34 (17.8)	0.861
Pre-operative IABP	8 (8.1)	9 (9.7)	17	0.405

COPD: chronic obstructive pulmonary disease, CRF: chronic renal failure, HT: hypertension, DM: diabetes mellitus, CVA: cerebrovascular accident, MI: myocardial infarction, BMI: body mass index, LMCA: left main coronary artery disease, EF: ejection fraction.

All of the patients were operated on with standard cardiopulmonary bypass under general anaesthesia. Antegrade cardioplegia was used for cardiac protection. In all cases, an IABP catheter was inserted through the common femoral artery. In this study, IABP was used intra-operatively when weaning from cardiopulmonary bypass had failed. Pre-operative IABP was used in cases of low cardiac output, unstable refractory angina, or persistent arrhythmia due to myocardial ischaemia.^5^,^10^

The patients were classified according to age; whether they were younger than 65 years or older. The mortality rate, complications of IABP, intra-operative properties, pre-operative clinical characteristics of patients, and length of stay in the intensive care unit were recorded.

The pre-operative parameters of the patients were age, gender, re-operation, hypertension, body mass index, diabetes mellitus, chronic renal failure, EuroSCORE value, previous cerebrovascular accident, left ventricular ejection fraction, left main coronary artery disease, chronic obstructive pulmonary disease (COPD), and the presence of a myocardial infarction more recent than one week earlier. The pre-operative clinical characteristics, postoperative complications, duration of ICU and hospital stays, and mortality rates of the groups were compared.

## Statistical analysis

Demographic characteristics were compared with mean and median values. Parametric results were evaluated using a Student’s *t*-test and Tukey test. A chi-square method, Pearson’s test, and Fisher’s test were used to analyse the categorical parameters. Risk factors for mortality were assessed using a binary logistic regression analysis. The standard deviation value *p* < 0.05 was considered significant. SPSS 18 was used for the statistical analysis.

## Results

In this study, 138 of the 190 patients were male. The mean patient age was 62.7 ± 9.9 years. Ninety-eight patients were younger than 65 years of age, and 90 patients were 65 years of age or older.

The number of patients with chronic obstructive pulmonary disease and the mean EuroSCORE value of the patients were higher in the older group. By contrast, the number of patients with diabetes mellitus was higher in the younger group. In terms of other demographic characteristics, there were no statistically significant differences between the groups (Table 1). The mean cardiopulmonary bypass times, mean cross-clamp times, and number of grafts used were similar between the two groups (Table 2).

**Table 2:** Mortality rates and clinical outcomes of the patients

	*Younger group (n = 98)*	*Older group (n = 92)*	*p-value*
Mortality	23 (23.4)	34 (36.9)	0.043
Mortality*	8 (44.4)	7 (41.1 %)	0.964
Mortality**	15 (18.7)	27 (36%)	0.018
CPB time (min)	143 ± 59	140 ± 58	0.786
Graft number	3.1 (2–5)	3.2 (2–5)	0.789
Cross clamp time (min)	90 ± 34	88 ± 38	0.604
ICU time (days)	5.9 ± 4	6.6 ± 5	0.284

ICU: intensive care unit, CPB time: cardiopulmonary bypass time, *patients undergoing emergency operations, **patients undergoing elective operations.

Fifty-seven (30.1%) patients died in the first 30 days following the operation. Twenty-three of these patients were in the younger group. The mortality rate of the younger group was significantly lower compared with the older patients (*p* = 0.043). In the subgroup analysis, the mortality rate of emergent operations was similar in the younger and older groups (*p* = 0.964). However, the mortality rate was higher in the older group for elective operations (*p* = 0.018).

Among the surviving patients, the number of older patients, rate of emergency operations, mean EuroSCORE values, and number of patients with chronic renal failure were lower than in the group of patients who died (Table 3). Binary logistic regression analysis showed that the only factor affecting mortality was prolonged cardiopulmonary bypass time. However, in the subgroup analysis of patients without emergency conditions, age was the second determinant of mortality (*p* = 0.018, OR = 5.5).

**Table 3:** Risk factors in patients who survived or died

	*Patients survived (n = 133)*	*Patients died (n = 57)*	*p-value*
Pre-operative MI,*n* (%)	40 (30)	15 (26.3)	0.601
BMI	27.5 ± 4.2	26.9 ± 4	0.507
EuroSCORE	4.2 (0-10)	5.1 (0-10)	0.030
DM, *n* (%)	47 (35.3)	24 (42.1)	0.377
CRF, *n* (%)	3 (2.2)	5 (8.7)	0.040
Mean EF %	38.4 ± 8	37.5 ± 9	0.562
Mean age (year)	61.8 ± 9.8	64.9 ± 10	0.051
Older patients, *n* (%)	58 (43.6)	34 (59.6)	0.043
Gender (female/male)	33/101	20/37	0.118
COPD, *n* (%)	12 (9)	6 (10.5)	0.746
Emergency operation, *n* (%)	19 (14.2)	15 (26.3)	0.047
LMCA, *n* (%)	8 (6)	5 (8.7)	0.490
CVA, *n* (%)	5 (3.7)	4 (7)	0.333
HT, *n* (%)	69 (51.8)	34 (59.6)	0.328
Re-operation, *n* (%)	3 (2.2)	0	0.555
Pre-operative IABP, *n* (%)	14 (10.5)	3 (2.2)	0.405
CPB time (min)	130 ± 48	167 ± 72	< 0.001
Cross-clamp time (min)	87 ± 35	94 ± 36	0.180

CPB time: cardiopulmonary bypass time. COPD: chronic obstructive pulmonary disease. CRF: chronic renal failure. HT: hypertension. DM: diabetes mellitus. CVA: previous cerebrovascular accident. BMI: body mass index. LMCA: left main coronary artery disease.

In the subgroup analysis, cardiopulmonary bypass time and pre-operative chronic renal failure were independent risk factors for mortality in the older group. In the younger group, female gender, diabetes mellitus, high EuroSCORE, emergency operation, prolonged cardiopulmonary bypass time (*p* = 0.001, OR = 7.6), and prolonged stay in the intensive care unit were independent risk factors for mortality (Table 4).

**Table 4:** Risk factors for mortality in subgroup analysis

	*Younger group*		*Older group*	
	*OR*	*p-value*	*OR*	*p-value*
COPD	0.035	0.851	0.015	0.903
CRF	0.168	0.682	4.205	0.040
Re-operation	0.949	0.330	-	-
EF (%)	0.865	0.352	0.110	0.759
Age (year)	0.122	0.727	1.034	0.741
EuroSCORE	14.555	0.000	8.418	0.309
CPB time (min)	7.698	0.006	0.471	0.004
Cross-clamp time (min)	2.048	0.152	1.542	0.493
BMI	0.703	0.402	0.384	0.214
Emergency operation	5.401	0.020	0.400	0.536
Female gender	8.850	0.003	1.725	0.527
HT	2.007	0.157	0.095	0.189
MI	0.427	0.513	0.004	0.758
DM	7.477	0.006	0.560	0.949
ICU time	4.947	0.026	0.038	0.454
Levosimendan	0.228	0.633	0.131	0.845
CVA	1.634	0.201	0.021	0.717
LMCA	0.955	0.329	0.021	0.885

CPB time: cardiopulmonary bypass time. COPD: chronic obstructive pulmonary disease. CRF: chronic renal failure. HT: hypertension. DM: diabetes mellitus. ICU: intensive care unit. CVA: previous cerebrovascular accident. BMI: body mass index. LMCA: left main coronary artery disease.

In our study, a few serious complications were observed due to IABP support. Iliac artery injury occurred in two patients and peripheral ischaemia was observed in three patients. The other complications were thrombocytopaenia and minor bleeding at the catheter site (Table 5). The rate of complications was similar between the groups.

**Table 5:** IABP complications according to patient groups

	*Younger group*	*Older group*	*p-value*
Bleeding, *n* (%)	1 (1)	4 (4.3)	0.200
Arterial injury, *n* (%)	0	2 (2.1)	0.233
Mild thrombocytopaenia, *n* (%)	10 (10.2)	15 (16.3)	0.309
Extremity ischaemia, *n* (%)	1 (1)	2 (2.1)	0.611
Total, *n* (%)	12 (12.2)	23 (25)	0.023

## Discussion

Postoperative recovery in elderly patients takes a longer time than in younger patients. Postoperative atrial fibrillation requiring medical treatment, and other complications occur more frequently in the elderly; the total intubation time is also longer for this group. Therefore, delayed recovery in the elderly may simply be due to the aging process affecting all organs.^9^ For this reason, elderly patients may need more mechanical support in cases of low cardiac output following cardiopulmonary bypass.

In the present study, while the number of COPD patients was higher in the older group, the number of diabetes mellitus patients was lower in the older group. In addition, EuroSCORE values were higher in the elderly patients. The mortality rate was higher in elderly patients; however, there were no statistically significant differences between the patients who had emergency surgery in both groups.

It has been reported that IABP decreases the mortality rates of low-cardiac output and severe myocardial ischaemia patients in the pre-operative period, provides support for patients who failed to wean from cardiopulmonary bypass during the intra-operative period, and prevents low cardiac output and medically refractory arrhythmias in intensive care units in the postoperative period.^11^,^12^ In this study, IABP was used in cases of low cardiac output, persistent angina pectoris, or arrhythmia due to myocardial ischaemia in the pre-operative period.

In previous studies, the use of pre-operative IABP in high-risk patients was reportedly more advantageous than peri-operative IABP support. Böning *et al.* compared the use of pre-operative and peri-operative IABP in high-risk patients in their study. Their results indicate that the pre-operative use of IABP was advantageous for early and long-term mortality.^13^ Dyub *et al.* showed that in a meta-analysis involving 1 034 patients, the use of pre-operative IABP in high-risk patients reduced mortality rates.^14^ Holman *et al.* reported that when shock, urgent surgery, haemodynamic instability, and MI in the last three days were excluded, the use of pre-operative IABP did not have a positive effect on morbidity and mortality rates; however, the length of the hospital stay was shorter in these patients.^15^

Miceli *et al.* proposed a scoring system that predicts the need for IABP support in high-risk coronary artery bypass patients.^16^ According to this study, heart failure, re-operation, emergency operation, left main coronary artery disease, patients over the age of 70 years, moderate and poor left ventricular function, and recent myocardial infarctions are independent risk factors for the need for IABP support. As a result of the study, the benefits of IABP support in patients with high-risk scores were emphasised. In our clinical practice, we did not use a risk-scoring system for prophylactic IABP support. In this study, we aimed to determine the pre-operative risk factors for mortality and other clinical outcomes.

In previous studies, emergency surgery, a history of myocardial infarction, prolonged cardiopulmonary bypass, and concomitant peripheral artery occlusive disease were all found to be significant determinants of mortality in primary isolated CABG patients.^17^ Furthermore, risk-scoring systems were generated. We showed that the mortality rate of the older patient group was higher than that in the younger group. However, the logistic regression analysis indicated that the only independent risk factor for mortality was a prolonged cardiopulmonary bypass time.

In addition, subgroup analysis revealed different results. For example, in the older patient group, chronic renal failure and prolonged cardiopulmonary bypass were identified as factors that affected mortality rate. In younger patients, female gender, diabetes mellitus, emergency operations, higher EuroSCORE values, prolonged cardiopulmonary bypass time, and prolonged stay in the ICU were independent risk factors for mortality. In elective operations, advanced patient age and prolonged cardiopulmonary bypass time were identified as factors that affected mortality rates.

Complications with the intra-aortic balloon pump were described in previous studies: limb ischaemia, thrombocytopaenia, arterial rupture or dissection, and sepsis and local infections.^4^-^6^,^10^,^18^ Complication rates have been reported from 26 to 50% in different studies. The risk factors for IABP complications were stated as increased age, female gender, duration of IABP treatment, presence of diabetes mellitus, and having several risk factors (e.g. obesity, smoking, hypertension, cardiogenic shock, inotropic support, low cardiac output, increased systemic vascular resistance, and ankle–brachial pressure index < 0.8).

In our study, the IABP complication rate was higher in older patients compared to younger patients (25 vs 12.2%). Mild thrombocytopaenia was the most frequently detected complication. When thrombocytopaenia is detected, IABP therapy is terminated immediately so that fewer bleeding complications occur.

## Limitations

Our study was a single-institution, retrospective study, which had a relatively small sample size. This subject may require further multicentre, randomised trials. Unaccounted for confounders may have been inherent in such a retrospective analysis.

## Conclusion

Intra-aortic balloon pumps are important cardiac support instruments that are easily implemented and have beneficial effects for resolving transient ischaemic situations. Whether young or old, patients who require IABP support have a high risk of mortality. Moreover, the association of elderly patients with increased incidences of co-morbid disease makes them even more susceptible to mortality. We question whether IABP may rather be used in the intra-operative period as a prophylactic device in elderly patients with multiple risk factors.
